# Supplementary figures and images for: The relationship between allergic rhinitis and attention deficit hyperactivity disorder: a systematic review and meta-analysis
Source: PeerJ. 2024 Oct 18;12:e18287. doi: 10.7717/peerj.18287 (PMC11493030; doi:10.7717/peerj.18287)

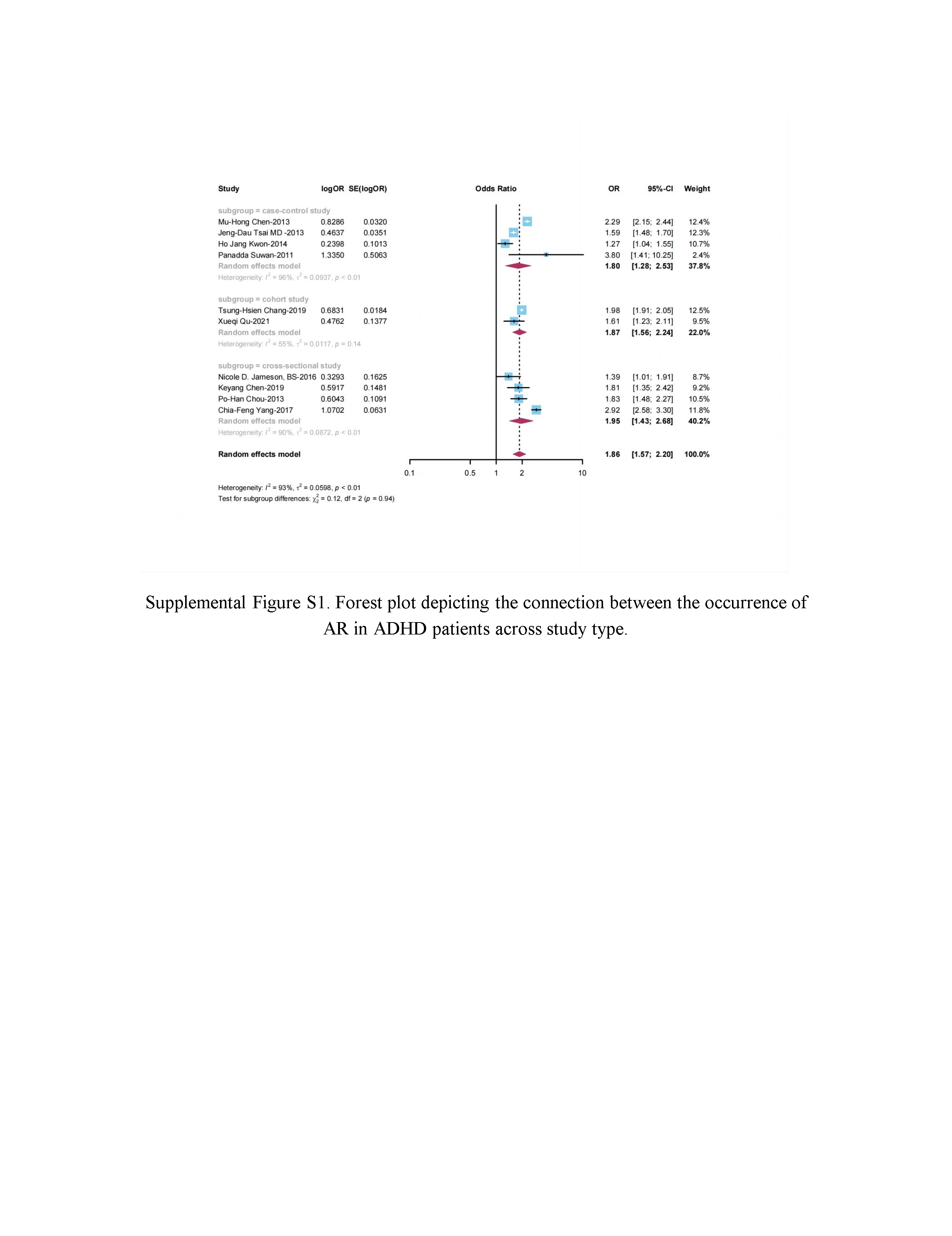

Supplement: Supplemental Information 2 [file peerj-12-18287-s002.jpg]

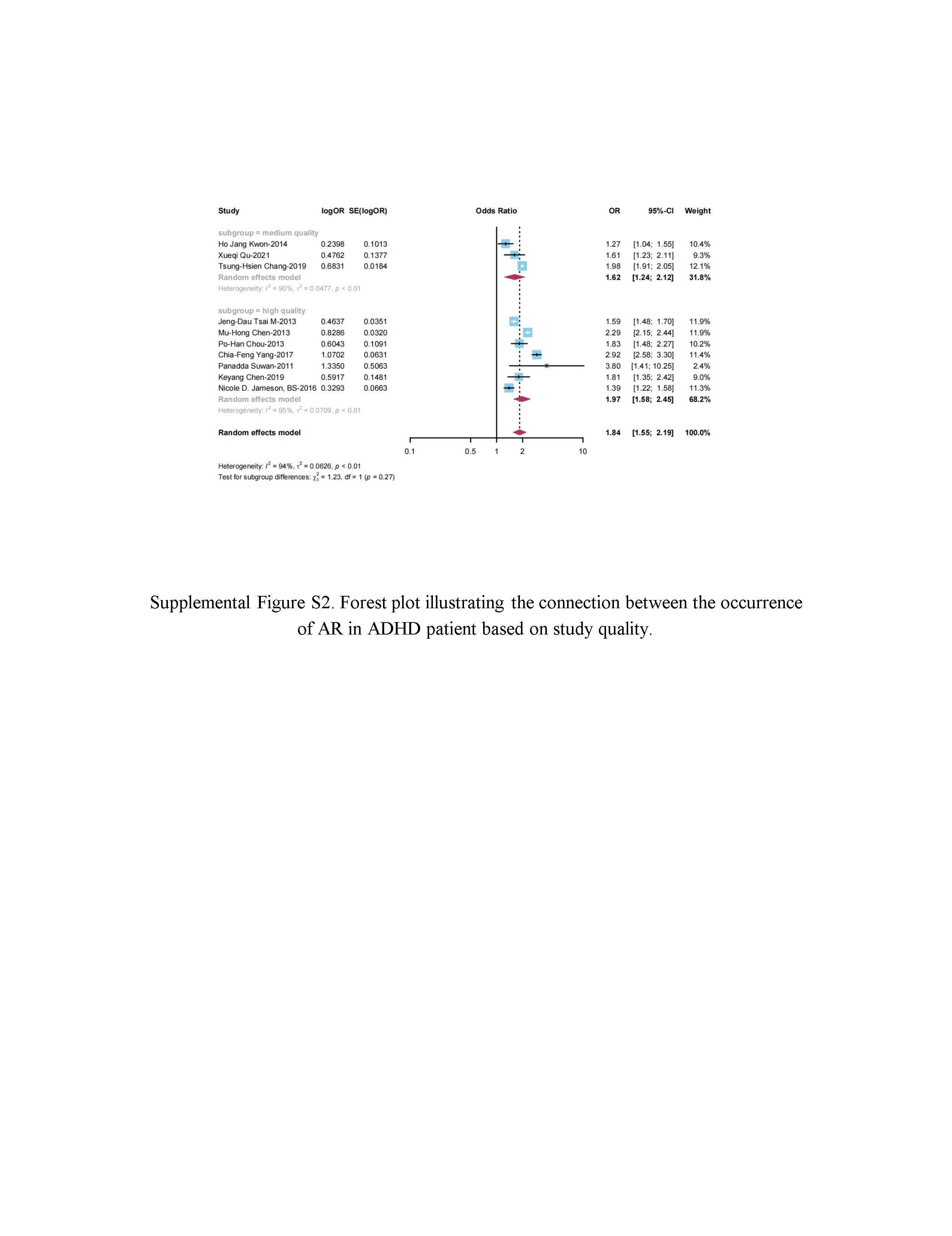

Supplement: Supplemental Information 3 [file peerj-12-18287-s003.jpg]

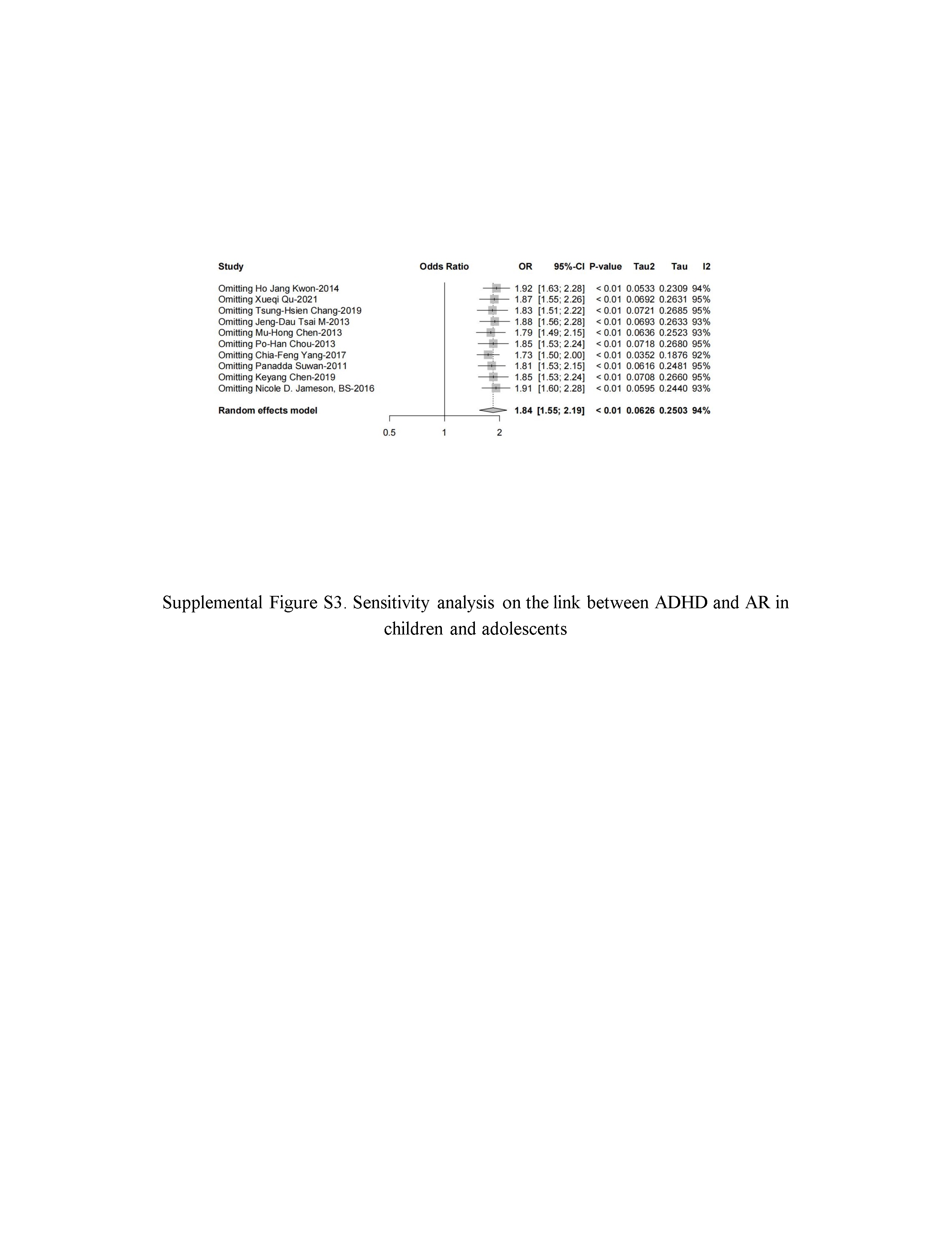

Supplement: Supplemental Information 4 [file peerj-12-18287-s004.jpg]

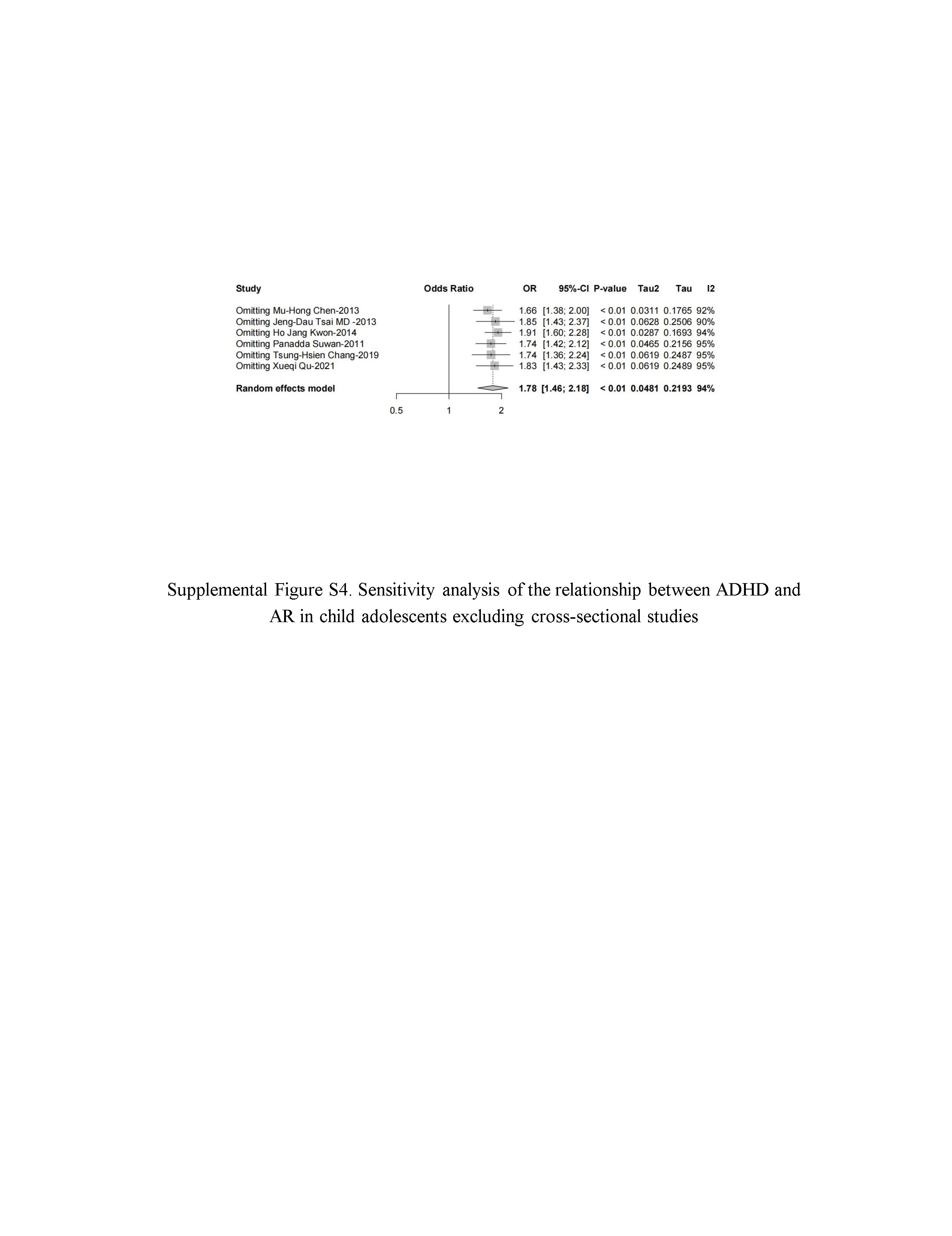

Supplement: Supplemental Information 5 [file peerj-12-18287-s005.jpg]
