# Supplementary material for: The relationship between allergic rhinitis and attention deficit hyperactivity disorder: a systematic review and meta-analysis
Source: PeerJ. 2024 Oct 18;12:e18287. doi: 10.7717/peerj.18287 (PMC11493030; doi:10.7717/peerj.18287)
Supplement: Supplemental Information 6 [file peerj-12-18287-s006.docx]

| Database | Retrieval formula |
| --- | --- |
| Pubmed | (((Rhinitis, Allergic[MeSH Terms]) OR (Rhinitis, Allergic, Seasonal[MeSH Terms])) OR ((((((((((((((((((((Allergic Rhinitides [Title/Abstract]) OR ( Rhinitides, Allergic [Title/Abstract])) OR (Allergic Rhinitis[Title/Abstract])) OR (Seasonal Allergic Rhinitis[Title/Abstract])) OR (Allergic Rhinitides, Seasonal[Title/Abstract])) OR (Allergic Rhinitis, Seasonal[Title/Abstract])) OR (Rhinitides, Seasonal Allergic[Title/Abstract])) OR (Rhinitis, Seasonal Allergic[Title/Abstract])) OR (Seasonal Allergic Rhinitides[Title/Abstract])) OR (Pollen Allergy[Title/Abstract])) OR (Allergies, Pollen[Title/Abstract])) OR (Allergy, Pollen[Title/Abstract])) OR (Pollen Allergies[Title/Abstract])) OR (Pollinosis[Title/Abstract])) OR (Pollinoses[Title/Abstract])) OR (Hay Fever[Title/Abstract])) OR (Fever, Hay[Title/Abstract])) OR (Hayfever[Title/Abstract])) OR (nasal allergy[Title/Abstract])) OR (rhinallergosis[Title/Abstract]))) AND (("Attention Deficit Disorder with Hyperactivity"[Mesh]) OR ((((((((((((((((((((((((((ADHD[Title/Abstract]) OR (Attention Deficit Hyperactivity Disorder[Title/Abstract])) OR (Hyperkinetic Syndrome[Title/Abstract])) OR (Hyperkinetic Syndromes[Title/Abstract])) OR (Syndrome, Hyperkinetic[Title/Abstract])) OR (Syndromes, Hyperkinetic[Title/Abstract])) OR (Hyperkinetic disorder[Title/Abstract])) OR (Hyperactivity disorder[Title/Abstract])) OR (overactive child syndrome[Title/Abstract])) OR (Attention Deficit-Hyperactivity Disorder[Title/Abstract])) OR (Attention Deficit-Hyperactivity Disorders[Title/Abstract])) OR (Deficit-Hyperactivity Disorder, Attention[Title/Abstract])) OR (Deficit-Hyperactivity Disorders, Attention[Title/Abstract])) OR (Disorder, Attention Deficit-Hyperactivity[Title/Abstract])) OR (Disorders, Attention Deficit-Hyperactivity[Title/Abstract])) OR (ADDH[Title/Abstract])) OR (Attention Deficit Hyperactivity Disorders[Title/Abstract])) OR (Attention deficit disorder Hyperactivity[Title/Abstract])) OR (Attention Deficit Disorder[Title/Abstract])) OR (Attention Deficit Disorders[Title/Abstract])) OR (Deficit Disorder, Attention[Title/Abstract])) OR (Deficit Disorders, Attention[Title/Abstract])) OR (Disorder, Attention Deficit[Title/Abstract])) OR (Disorders, Attention Deficit[Title/Abstract])) OR (Brain Dysfunction, Minimal[Title/Abstract])) OR (Minimal Brain Dysfunction[Title/Abstract]))) |
| Ovid databases(Ovid MEDLINE) | (Rhinitis, Allergic OR Rhinitis, Allergic, Seasonal OR Allergic Rhinitides OR Rhinitides, Allergic OR Allergic Rhinitis OR Seasonal Allergic Rhinitis OR Allergic Rhinitides, Seasonal OR Allergic Rhinitis, Seasonal OR Rhinitides, Seasonal Allergic OR Rhinitis, Seasonal Allergic OR Seasonal Allergic Rhinitides OR Pollen Allergy OR Allergies, Pollen OR Allergy, Pollen OR Pollen Allergies OR Pollinosis OR Pollinoses OR Hay Fever OR Fever, Hay OR Hayfever OR nasal allergy OR rhinallergosis ) AND ( Attention Deficit Disorder with Hyperactivity OR ADHD OR Attention Deficit Hyperactivity Disorder OR Hyperkinetic Syndrome OR Hyperkinetic Syndromes OR Syndrome, Hyperkinetic OR Syndromes, Hyperkinetic OR Hyperkinetic disorder OR Hyperactivity disorder OR overactive child syndrome OR Attention Deficit-Hyperactivity Disorder OR Attention Deficit-Hyperactivity Disorders OR Deficit-Hyperactivity Disorder, Attention OR Deficit-Hyperactivity Disorders, Attention OR Disorder, Attention Deficit-Hyperactivity OR Disorders, Attention Deficit-Hyperactivity OR ADDH OR Attention Deficit Hyperactivity Disorders OR Attention deficit disorder Hyperactivity OR Attention Deficit Disorder OR Attention Deficit Disorders OR Deficit Disorder, Attention OR Deficit Disorders, Attention OR Disorder, Attention Deficit OR Disorders, Attention Deficit OR Brain Dysfunction, Minimal OR Minimal Brain Dysfunction) |
| Cochrane library | (((Rhinitis, Allergic[MeSH Terms]) OR (Rhinitis, Allergic, Seasonal[MeSH Terms])) OR ((((((((((((((((((((Allergic Rhinitides[Title/Abstract]) OR (Rhinitides, Allergic[Title/Abstract])) OR (Allergic Rhinitis[Title/Abstract])) OR (Seasonal Allergic Rhinitis[Title/Abstract])) OR (Allergic Rhinitides, Seasonal[Title/Abstract])) OR (Allergic Rhinitis, Seasonal[Title/Abstract])) OR (Rhinitides, Seasonal Allergic[Title/Abstract])) OR (Rhinitis, Seasonal Allergic[Title/Abstract])) OR (Seasonal Allergic Rhinitides[Title/Abstract])) OR (Pollen Allergy[Title/Abstract])) OR (Allergies, Pollen[Title/Abstract])) OR (Allergy, Pollen[Title/Abstract])) OR (Pollen Allergies[Title/Abstract])) OR (Pollinosis[Title/Abstract])) OR (Pollinoses[Title/Abstract])) OR (Hay Fever[Title/Abstract])) OR (Fever, Hay[Title/Abstract])) OR (Hayfever[Title/Abstract])) OR (nasal allergy[Title/Abstract])) OR (rhinallergosis[Title/Abstract]))) AND (("Attention Deficit Disorder with Hyperactivity"[Mesh]) OR ((((((((((((((((((((((((((ADHD[Title/Abstract]) OR (Attention Deficit Hyperactivity Disorder[Title/Abstract])) OR (Hyperkinetic Syndrome[Title/Abstract])) OR (Hyperkinetic Syndromes[Title/Abstract])) OR (Syndrome, Hyperkinetic[Title/Abstract])) OR (Syndromes, Hyperkinetic[Title/Abstract])) OR (Hyperkinetic disorder[Title/Abstract])) OR (Hyperactivity disorder[Title/Abstract])) OR (overactive child syndrome[Title/Abstract])) OR (Attention Deficit-Hyperactivity Disorder[Title/Abstract])) OR (Attention Deficit-Hyperactivity Disorders[Title/Abstract])) OR (Deficit-Hyperactivity Disorder, Attention[Title/Abstract])) OR (Deficit-Hyperactivity Disorders, Attention[Title/Abstract])) OR (Disorder, Attention Deficit-Hyperactivity[Title/Abstract])) OR (Disorders, Attention Deficit-Hyperactivity[Title/Abstract])) OR (ADDH[Title/Abstract])) OR (Attention Deficit Hyperactivity Disorders[Title/Abstract])) OR (Attention deficit disorder Hyperactivity[Title/Abstract])) OR (Attention Deficit Disorder[Title/Abstract])) OR (Attention Deficit Disorders[Title/Abstract])) OR (Deficit Disorder, Attention[Title/Abstract])) OR (Deficit Disorders, Attention[Title/Abstract])) OR (Disorder, Attention Deficit[Title/Abstract])) OR (Disorders, Attention Deficit[Title/Abstract])) OR (Brain Dysfunction, Minimal[Title/Abstract])) OR (Minimal Brain Dysfunction[Title/Abstract]))) |
| EMBASE | (Rhinitis, Allergic OR Rhinitis, Allergic, Seasonal OR Allergic Rhinitides OR Rhinitides, Allergic OR Allergic Rhinitis OR Seasonal Allergic Rhinitis OR Allergic Rhinitides, Seasonal OR Allergic Rhinitis, Seasonal OR Rhinitides, Seasonal Allergic OR Rhinitis, Seasonal Allergic OR Seasonal Allergic Rhinitides OR Pollen Allergy OR Allergies, Pollen OR Allergy, Pollen OR Pollen Allergies OR Pollinosis OR Pollinoses OR Hay Fever OR Fever, Hay OR Hayfever OR nasal allergy OR rhinallergosis ) AND ( Attention Deficit Disorder with Hyperactivity OR ADHD OR Attention Deficit Hyperactivity Disorder OR Hyperkinetic Syndrome OR Hyperkinetic Syndromes OR Syndrome, Hyperkinetic OR Syndromes, Hyperkinetic OR Hyperkinetic disorder OR Hyperactivity disorder OR overactive child syndrome OR Attention Deficit-Hyperactivity Disorder OR Attention Deficit-Hyperactivity Disorders OR Deficit-Hyperactivity Disorder, Attention OR Deficit-Hyperactivity Disorders, Attention OR Disorder, Attention Deficit-Hyperactivity OR Disorders, Attention Deficit-Hyperactivity OR ADDH OR Attention Deficit Hyperactivity Disorders OR Attention deficit disorder Hyperactivity OR Attention Deficit Disorder OR Attention Deficit Disorders OR Deficit Disorder, Attention OR Deficit Disorders, Attention OR Disorder, Attention Deficit OR Disorders, Attention Deficit OR Brain Dysfunction, Minimal OR Minimal Brain Dysfunction) |
| CINAHL | TX ( Rhinitis, Allergic OR Rhinitis, Allergic, Seasonal OR Allergic Rhinitides OR Rhinitides, Allergic OR Allergic Rhinitis OR Seasonal Allergic Rhinitis OR Allergic Rhinitides, Seasonal OR Allergic Rhinitis, Seasonal OR Rhinitides, Seasonal Allergic OR Rhinitis, Seasonal Allergic OR Seasonal Allergic Rhinitides OR Pollen Allergy OR Allergies, Pollen OR Allergy, Pollen OR Pollen Allergies OR Pollinosis OR Pollinoses OR Hay Fever OR Fever, Hay OR Hayfever OR nasal allergy OR rhinallergosis ) AND TX ( Attention Deficit Disorder with Hyperactivity OR ADHD OR Attention Deficit Hyperactivity Disorder OR Hyperkinetic Syndrome OR Hyperkinetic Syndromes OR Syndrome, Hyperkinetic OR Syndromes, Hyperkinetic OR Hyperkinetic disorder OR Hyperactivity disorder OR overactive child syndrome OR Attention Deficit-Hyperactivity Disorder OR Attention Deficit-Hyperactivity Disorders OR Deficit-Hyperactivity Disorder, Attention OR Deficit-Hyperactivity Disorders, Attention OR Disorder, Attention Deficit-Hyperactivity OR Disorders, Attention Deficit-Hyperactivity OR ADDH OR Attention Deficit Hyperactivity Disorders OR Attention deficit disorder Hyperactivity OR Attention Deficit Disorder OR Attention Deficit Disorders OR Deficit Disorder, Attention OR Deficit Disorders, Attention OR Disorder, Attention Deficit OR Disorders, Attention Deficit OR Brain Dysfunction, Minimal OR Minimal Brain Dysfunction ) |
| Web of science | (TS=(Rhinitis, Allergic OR Rhinitis, Allergic, Seasonal OR Allergic Rhinitides OR Rhinitides, Allergic OR Allergic Rhinitis OR Seasonal Allergic Rhinitis OR Allergic Rhinitides, Seasonal OR Allergic Rhinitis, Seasonal OR Rhinitides, Seasonal Allergic OR Rhinitis, Seasonal Allergic OR Seasonal Allergic Rhinitides OR Pollen Allergy OR Allergies, Pollen OR Allergy, Pollen OR Pollen Allergies OR Pollinosis OR Pollinoses OR Hay Fever OR Fever, Hay OR Hayfever OR nasal allergy OR rhinallergosis )) AND TS=(Attention Deficit Disorder with Hyperactivity OR ADHD OR Attention Deficit Hyperactivity Disorder OR Hyperkinetic Syndrome OR Hyperkinetic Syndromes OR Syndrome, Hyperkinetic OR Syndromes, Hyperkinetic OR Hyperkinetic disorder OR Hyperactivity disorder OR overactive child syndrome OR Attention Deficit-Hyperactivity Disorder OR Attention Deficit-Hyperactivity Disorders OR Deficit-Hyperactivity Disorder, Attention OR Deficit-Hyperactivity Disorders, Attention OR Disorder, Attention Deficit-Hyperactivity OR Disorders, Attention Deficit-Hyperactivity OR ADDH OR Attention Deficit Hyperactivity Disorders OR Attention deficit disorder Hyperactivity OR Attention Deficit Disorder OR Attention Deficit Disorders OR Deficit Disorder, Attention OR Deficit Disorders, Attention OR Disorder, Attention Deficit OR Disorders, Attention Deficit OR Brain Dysfunction, Minimal OR Minimal Brain Dysfunction) |
| ERIC | (Rhinitis, Allergic OR Rhinitis, Allergic, Seasonal OR Allergic Rhinitides OR Rhinitides, Allergic OR Allergic Rhinitis OR Seasonal Allergic Rhinitis OR Allergic Rhinitides, Seasonal OR Allergic Rhinitis, Seasonal OR Rhinitides, Seasonal Allergic OR Rhinitis, Seasonal Allergic OR Seasonal Allergic Rhinitides OR Pollen Allergy OR Allergies, Pollen OR Allergy, Pollen OR Pollen Allergies OR Pollinosis OR Pollinoses OR Hay Fever OR Fever, Hay OR Hayfever OR nasal allergy OR rhinallergosis ) AND ( Attention Deficit Disorder with Hyperactivity OR ADHD OR Attention Deficit Hyperactivity Disorder OR Hyperkinetic Syndrome OR Hyperkinetic Syndromes OR Syndrome, Hyperkinetic OR Syndromes, Hyperkinetic OR Hyperkinetic disorder OR Hyperactivity disorder OR overactive child syndrome OR Attention Deficit-Hyperactivity Disorder OR Attention Deficit-Hyperactivity Disorders OR Deficit-Hyperactivity Disorder, Attention OR Deficit-Hyperactivity Disorders, Attention OR Disorder, Attention Deficit-Hyperactivity OR Disorders, Attention Deficit-Hyperactivity OR ADDH OR Attention Deficit Hyperactivity Disorders OR Attention deficit disorder Hyperactivity OR Attention Deficit Disorder OR Attention Deficit Disorders OR Deficit Disorder, Attention OR Deficit Disorders, Attention OR Disorder, Attention Deficit OR Disorders, Attention Deficit OR Brain Dysfunction, Minimal OR Minimal Brain Dysfunction) |
|  |  |

# Supplemental Table S1. Search terms for each electronic database
