# Supplementary material for: The relationship between allergic rhinitis and attention deficit hyperactivity disorder: a systematic review and meta-analysis
Source: PeerJ. 2024 Oct 18;12:e18287. doi: 10.7717/peerj.18287 (PMC11493030; doi:10.7717/peerj.18287)
Supplement: Supplemental Information 7 [file peerj-12-18287-s007.docx]

| Cross-sectional study | Score | | | |
| --- | --- | --- | --- | --- |
| items | Chen et al (2019) | Chou et al (2013) | Jameson et al (2016) | Yang et al (2017) |
| Define the source of information (survey, record review) | Yes | Yes | Yes | Yes |
| List inclusion and exclusion criteria for exposed and unexposed subjects (cases and controls) or refer to previous publications | No | Yes | No | Yes |
| Indicate the time period used for identifying patients | Yes | No | No | No |
| Indicate whether or not subjects were consecutive if not population-based | Yes | Yes | Yes | Yes |
| Indicate if evaluators of subjective components of study were masked to other aspects ofthe status of the participants | Unclear | Unclear | Unclear | No |
| Describe any assessments undertaken for quality assurance purposes (e.g., test/retest ofprimary outcome measurements ) | No | No | No | Yes |
| Explain any patient exclusions from analysis | Yes | Yes | No | Yes |
| Describe how confounding was assessed and/or controlled | Unclear | Yes | Yes | Yes |
| lf applicable, explain how missing data were handled in the analysis | No | No | No | No |
| Summarize patient response rates and completeness of data collection | Yes | Yes | Yes | Yes |
| Clarify what follow-up, if any, was expected and the percentage of patients for which incomplete data or follow-up was obtained | No | No | No | No |
| Total Score | 5 | 6 | 4 | 6 |

| CCase-control study | Score | | | | |
| --- | --- | --- | --- | --- | --- |
| items | Suwan et al (2011) | Tsai et al (2013) | Lee et al (2014) | Kwon et al (2014) | Chen et al (2013) |
| Is the case definition adequate? | *(With independent verification) Diagnosed twice based on SPT) | *(With linkage data ICD-9-CM) | *(Diagnosed by a specialist) | *(Using ICD Diagnosis) * (Using Epidemic Survey Questionnaire+TOVA+CPRS+DuPaul ADHD) | *(NHIRD)+* (Doctor's diagnosis) |
| Representativeness of the cases | *(Diagnosed by hospital doctors) | *(Two year outpatient diagnosis) | *(Hospital outpatient recruitment) Recruitment time and quantity? | *(Seven regions in Shanghai) | *(NHIRD) |
| Selection of Controls | Hospital control (outpatient recruitment) | Hospital control | Hospital control | *Community control | Hospital control |
| Definition of Controls | *(Non patient) | *(Non patient) | *(Non patient) | *(Non patient) | *(Non patient) |
| Comparability of cases and controls on the basis of the design or analysis | *(controlled for age and gender) * (also matched with family history and living environment) | *Matching age, gender, indicator year, and indicator month * (controlling for confounding urbanization levels) | *(Age and gender matching) | *(Age and gender matching) | *(Age and gender matching) |
| Ascertainment of exposure | In a non blind setting (knowing who is in the case group and who is in the control group) | Non blind (identifying case groups and recruited control groups in the database) | Non blind | *(In the blind method: after distributing the survey questionnaire, it is separated) | Non blind |
| Same method of ascertainment for cases and controls | *Yes (SPT) | *Yes (SPT) | *Yes (SPT) | *Yes (DSM) | *Yes (NHIRD) |
| Non-Response rate | No Description | No Description | No Description | No Description | No Description |
| Total Score | 6 | 6 | 5 | 8 | 6 |

| Cohort study | Score | | |
| --- | --- | --- | --- |
| Items | Nemet et al (2022) | Chang et al (2019) | Qu et al (2021) |
| Representative of the exposed cohort | *The Clalit Health Services database (the largest pediatric database in Israel) | *NHIRD | *Participants in the Boston birth queue |
| Selection of the non-exposed cohort | From the same database above | From the same database above | Unspecified |
| Ascertainment of exposure | *Determine based on ICD-9 diagnostic code | *ICD-9-M | *ICD |
| Demonstration that outcome of interest was not present at the start of the study | *Yes | *Yes | *Yes |
| Comparability of cohorts on the basis of the design or analysis | *Age matching | **Age, gender, time of birth, and place of residence | **Age, gender, race, family history |
| Assessment of outcome | **At least two medical records and diagnosis based on ICD and DSM | *Doctor ICD diagnosis | *Doctor ICD diagnosis |
| Was follow-up long enough for outcomes to occur | *Yes | *Yes | *Yes |
| Adequacy of follow-up of cohorts | Undeclared | Undeclared | Undeclared |
| Total Score | 8* | 8* | 7* |

# Supplemental Table S2. Research Quality Rating Scale
